# Withdrawal ruptures in adolescents with borderline personality disorder psychotherapy are marked by increased speech pauses–can minimal responses be automatically detected?

**DOI:** 10.1371/journal.pone.0280329

**Published:** 2023-01-17

**Authors:** Christophe Künsch, Lukas Fürer, Martin Steppan, Nathalie Schenk, Kathrin Blum, Michael Kaess, Julian Koenig, Klaus Schmeck, Ronan Zimmermann

**Affiliations:** 1 Faculty of Psychology, University of Basel, Basel, Switzerland; 2 Child and Adolescent Psychiatric Research Department, Psychiatric University Hospital Basel, Basel, Switzerland; 3 Department of Child and Adolescent Psychiatry, Centre of Psychosocial Medicine, University of Heidelberg, Heidelberg, Germany; 4 University Hospital of Child and Adolescent Psychiatry and Psychotherapy, University of Bern, Bern, Switzerland; 5 Department of Child and Adolescent Psychiatry, Psychosomatics and Psychotherapy, Faculty of Medicine and University Hospital Cologne, University of Cologne, Cologne, Germany; Medical University of Vienna, AUSTRIA

## Abstract

Alliance ruptures of the withdrawal type are prevalent in adolescents with borderline personality disorder (BPD). Longer speech pauses are negatively perceived by these patients. Safran and Muran’s rupture model is promising but its application is very work intensive. This workload makes research costly and limits clinical usage. We hypothesised that pauses can be used to automatically detect one of the markers of the rupture model i.e. the minimal response marker. Additionally, the association of withdrawal ruptures with pauses was investigated. A total of 516 ruptures occurring in 242 psychotherapy sessions collected in 22 psychotherapies of adolescent patients with BPD and subthreshold BPD were investigated. Trained observers detected ruptures based on video and audio recordings. In contrast, pauses were automatically marked in the audio-recordings of the psychotherapy sessions and automatic speaker diarisation was used to determine the speaker-switching patterns in which the pauses occur. A random forest classifier detected time frames in which ruptures with the minimal response marker occurred based on the quantity of pauses. Performance was very good with an area under the ROC curve of 0.89. Pauses which were both preceded and followed by therapist speech were the most important predictors for minimal response ruptures. Research costs can be reduced by using machine learning techniques instead of manual rating for rupture detection. In combination with other video and audio derived features like movement analysis or automatic facial emotion detection, more complete rupture detection might be possible in the future. These innovative machine learning techniques help to narrow down the mechanisms of change of psychotherapy, here specifically of the therapeutic alliance. They might also be used to technologically augment psychotherapy training and supervision.

## 1. Introduction

Therapeutic alliance has been identified as one of the most robust predictors for therapy outcome [1, 2]. Alliance-outcome correlations are of moderate effect size [3–5]. According to the rupture and resolution model [6], the therapeutic alliance is dynamic and it is constantly negotiated between the patient and the therapist. "An alliance rupture is [defined as] a deterioration in the alliance, manifested by a lack of collaboration between patient and therapist on tasks or goals, or a strain in the emotional bond.” [7, p. 2]. Ruptures are conceptualised as inevitable in psychotherapy and offer windows of opportunity for the therapeutic process [8]. Two types of ruptures are distinguished by the model: ‘withdrawal ruptures’, in which the patient is, moving away” from the therapist, and ‘confrontation ruptures’, in which the patient, moves against” the therapist [9–12]. According to this model, the therapist can use rupture-resolution-strategies to repair ruptures, which maintains and strengthens the therapist-patient bond. Examples of resolution strategies are the reconceptualization of the patient’s task or disclosing the therapist’s perception of the rupture. The resolution process is thought to be therapeutic in itself [6].

Borderline personality disorder (BPD) is characterized by persistent patterns of i.a. unstable and intense personal relationships and fear of abandonment [13]. The disorder is known to impact the therapeutic alliance [14, 15]. Consequently, a good management of the therapeutic relationship is of high importance in the psychotherapy of these patients [15]. A dynamic conceptualisation of alliance as offered by the rupture and resolution model is of theoretical and, likely, of practical interest for the treatment of BPD. Research data supports the idea that personality disorders, in general, are associated with a higher number of ruptures. However, the evidence base is somewhat inconclusive [16]. It has been hypothesised that specific rupture training can improve therapy outcome, however, this association was not statistically confirmed [17].

A growing body of research shows the importance of early detection and intervention in BPD already in youth [18] and, as a consequence, the age threshold for diagnosing personality disorders has been omitted in the ICD-11 [19]. Our research group has studied the effectiveness of Adolescent Identity Treatment (AIT) and Dialectic Behaviour Therapy in adolescent patients with BPD. The result of this study showed that both these treatments are effective at improving psychosocial functioning and personality functioning in these patients [20]. In the same project [21], we have also studied ruptures and resolutions occurring in the psychotherapy with AIT. This research was done based on video recordings of the psychotherapeutic sessions which were reviewed by trained observers and rated with the ‘Rupture and Resolution Rating System’ (3RS) [7]. The resulting paper by Schenk et al. [22] focused on the trajectories of ruptures over entire psychotherapies. Withdrawal ruptures occurred more often than confrontation ruptures. Most ruptures occurred in the middle of the treatment, and, additionally, alliance struggle peaks could be identified, mostly after an impactful rupture [22]. The most frequent rupture marker was “minimal response” (please see below for a definition). Confrontation ruptures seemed to have a stronger impact than withdrawal ruptures [22]. A major limitation of the study by Schenk et al. [22] was that it only contained 10 fully analysed psychotherapies while our total sample amounted to 23 cases [20]. This was due to a lack of 3RS trained personnel since the involved PhD and master students finished their projects. After the initial 10 cases, the decision was made to only rate the initial 5 sessions of each psychotherapy to answer questions on early alliance.

While clinically very insightful and popular over the last years, there are still many open questions regarding ruptures and resolutions and their usefulness in the treatment of personality disorders. As the model entails the careful observation of psychotherapeutic sessions on a moment to moment basis [22–25], its application is very resource intensive. In addition, the observers need to be trained and inter-rater reliability needs to be ensured to allow for replicability. This can be difficult to maintain over time due to changes in personnel. The resource intensity is a barrier for research, e.g., producing adequately powered research while observing the dynamic of the alliance over full therapies will be very expensive. Additionally, the translation of the model into clinical practice, where resources are even scarcer, is highly problematic, if not impossible. With this in mind, while not part of our initial research protocols [21, 26], our research group has gained interest in automated evaluations of psychotherapy sessions based on audio and video recordings (e.g., dyadic speech pattern analysis or facial emotion recognition) and used these techniques in the sample that will be investigated in the current study [27–30]. These techniques allow for the standardised processing of entire psychotherapies within hours, minutes or even in real time. From a feasibility perspective, these automated methods have a much greater potential to translate into clinical practice, if they are proven clinically useful. While a real-time detection during the psychotherapy might not be desirable, some researchers see potential in digital tools that can support quality assurance of psychotherapy for training and supervision. As an example, a web-based platform which organises session recordings and, according to the creators of the platform, provides clinically relevant markers based on audio recordings, has already been developed [31].

Consequently, such technology-based procedures might potentially be used to automatically detect ruptures rendering research with this model much more affordable and offering a perspective for clinical implementations. Such a detection has been piloted [32]: Dolev-Amit et al. argue that the detection of withdrawal ruptures is critical as these ruptures often go unnoticed. They hypothesised that acoustic data could serve as a marker for withdrawal ruptures. Dolev-Amit et al. were able to show, in a case study, that a higher- F0 span, speech pause proportion and shimmer as well as a lower articulation rate than neutral speech can be used to mark withdrawal ruptures. The in-depth case study allowed them to draw up a scenario in which previously missed ruptures are identified during supervision. They also discussed the future potential of such markers to be used in real time computer assisted psychotherapy.

In the current study, we were specifically interested in using speech pauses as a predictor and investigate their correlation with ruptures. This interest was motivated by our previous study on speech pauses (silence) conducted on the sample used in the current article [27]. The study investigated the correlation of speech pauses with post session evaluations of the “smoothness” and “goodness” of the overall session (measured with the Session Evaluation Questionnaire [33, 34]). Sessions with more pauses were perceived as worse and less smooth by the adolescent patients with BPD, making pauses potentially problematic. Additionally, we had used automatic speaker diarisation (analysing who speaks when) to investigate the effect of the four possible speaker switching patterns in which pauses can be located (e.g., the pause could be located between two therapist speaking turns or, alternatively, between a patient speaking turn and a therapist speaking turn, …). In this session level analysis, the amount of pauses in the different speaker switching patterns was highly intercorrelated and therefore aggregated for the analysis. However, we concluded that future research should correlate pauses with “significant therapeutic events such as ruptures” [27, p. 167]. One of the aims of the current study is to follow-up on this research suggestion. First, we hypothesised that ruptures in general would contain more pauses than non-ruptures (H1). Second, we hypothesised that withdrawal ruptures would be characterised by more pauses than confrontation ruptures (H2).

In addition, we hypothesised that automatically detected pauses would, to a certain extent, allow for an automatic detection of rupture markers. Considering the results of the explorative case study by Dolev-Amit et al. [32], it makes sense to target withdrawal ruptures. However, the concept of withdrawal ruptures is an amalgam of multiple rupture markers (denial, minimal response, abstract communication, avoidant storytelling and shifting topic, deferential and appeasing) [7] making withdrawal ruptures a too complex and abstract construct to be targeted with automatic procedures. Instead, we targeted the “minimal response” marker. This rupture marker is defined as “withdrawal from the therapist by going silent or by giving minimal responses to questions or statements that are intended to initiate or continue discussion” [7]. The predictive counterpart in our hypothesised model, speech pause, has a more technical definition and is just absence of speech in a verbal interaction. The concepts withdrawal rupture and speech pause are, thus, similar but not congruent. A major difference is that, in the current context, minimal response markers are detected by a 3RS trained human who observes video material of psychotherapies and considers the overall therapeutic situation based on video and audio input. Pauses, in contrast, are, in the current context, automatically detected based on audio recordings. Based on the strong conceptual link, and the fact that minimal response is the most frequent rupture marker in adolescents with BPD, we aimed at automatically detecting minimal responses. We hypothesised that automatically detecting ruptures with “minimal response” markers based on speech pauses in different speaker switching patterns would be feasible (H3). For a clearer understanding of the study, it is noteworthy that the 3RS rating system acknowledges that the same sentence can be labelled as confrontation rupture and withdrawal rupture at the same time [7]. However, in these cases, we selected the dominant type to make rupture type (confrontation and withdrawal) mutually exclusive. Additionally, a rupture can have multiple rupture markers (e.g. minimal response, denial, complaints about the psychotherapist) which are not mutually exclusive. Minimal response markers which are associated with withdrawal ruptures could, thus, occur in segments with the dominant type ‘confrontation rupture’.

## 2. Materials and methods

The present study is part of the multi-centre study, Evaluation of Adolescent Identity Treatment” [20, 21, 35], registered at clinicaltrials.gov (NCT02518906). The results of the clinical trial have been published by Schmeck et al. [20]. The following analysis uses the available data registered at a single participating centre (Psychiatric University Hospitals of Basel). Ethical approval has been obtained from the local ethics committee (Ethikkommission Nordwest- und Zentralschweiz: Nr.: 2015–230). Written consent has been signed by participating adolescents, parents and therapists. The current study is a secondary analysis which combines data from two previous studies by Zimmermann et al. [27] and Schenk et al. [22].

### 2.1. Sample

**Fig 1:**
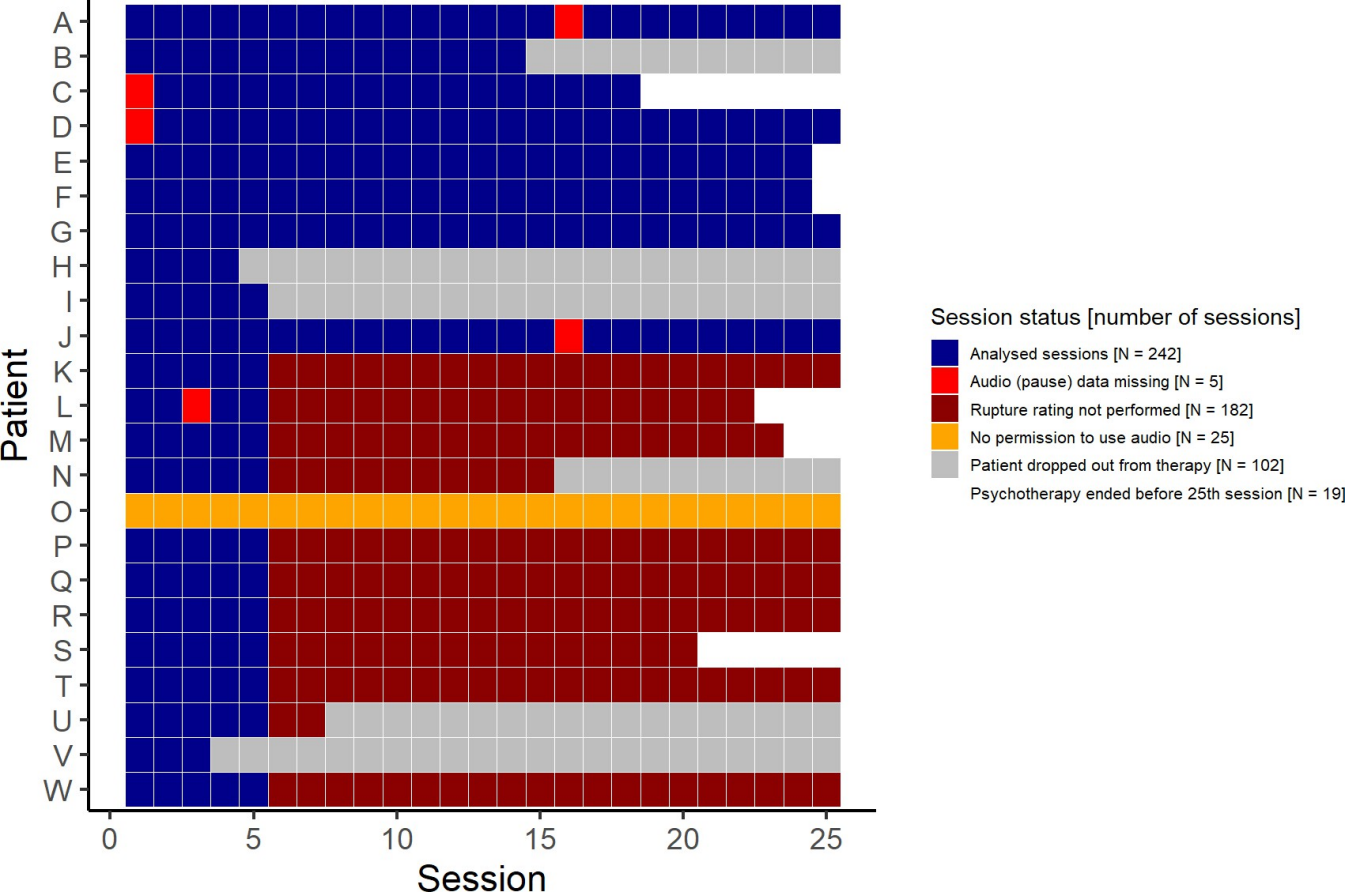
Analysed sessions and missing sessions.

The inclusion criteria for patients were: age between 13 and 19 years; at least three BPD criteria according to the Structured Clinical Interview For DSM-IV Axis II Personality Disorders (SCID-II); and identity diffusion (total *t-*score > 60), evaluated with the Assessment of Identity Development in Adolescence [36, 37]. The overall sample of 23 patients is described in the paper by Schmeck et al. [20] in Table 1. Only the patients in the AIT study arm were included here. Since one patient withdrew the permission to use the video material, the final sample comprised *N* = 22 patients. The mean age of the participants was 16.3 years (*SD =* 1.6). Six patients dropped out of treatment but the recorded sessions were included in the analysis. Fig 1 shows the available and missing sessions for each of the 23 patients. It also shows which sessions were missing for which reason.

**Table 1:** Speech pause percentage according to rupture type and minimal response markers.

*Rupture Type*	*Minimal Response*	*mean*	*sd*	*min*	*max*	*quantile 25%*	*median*	*quantile 75%*
Confrontation	No	32.05	21.29	0	100	16.00	28.00	44.00
Confrontation	Yes	45.01	25.04	0	100	26.00	42.00	60.00
Withdrawal	No	38.09	21.95	0	100	22.00	34.00	52.00
Withdrawal	Yes	48.86	25.69	0	100	28.00	46.00	68.00

The rupture and resolution rating used in the current study are re-used from a previous study by Schenk et al. [22]. As described in the introduction section, only the initial 10 patients had all psychotherapy sessions rated with the 3RS. Due to lack of personnel, only the five first sessions were rated for the subsequent 13 patients. Additionally, five records of therapeutic sessions could not be evaluated due to technical difficulties or human errors (i.e., the data was not saved). The analysed sample consisted of 242 psychotherapeutic sessions with a planned duration of 50 minutes per session. Eight psychotherapists were involved.

### 2.2. Setting and data acquisition

The included patients were treated with up to 25 session of AIT [38]. AIT is an integrative manualised psychotherapy approach for the treatment of adolescent personality disorder. It uses psychodynamic elements, psychoeducation and a cognitive-behaviour oriented home plan. AIT was shown to be equally effective compared to the more established treatment approach Dialectic Behaviour Therapy in adolescent patients within this clinical trial [20]. The sessions were video recorded with two cameras mounted to observe both the patient and therapist from the front. Audio was recorded with a boundary microphone attached to an adjacent wall between patient and therapist.

### 2.3. Speech pause detection

The audio recordings were cut to start at the beginning of the actual psychotherapeutic process (patient and therapist in sitting position and therapist invites patient to start the session) and end when the therapist formally ended the session. Silence detection was performed with the matlab code available in the repository ‘https://github.com/com-psy-lab/Silence-Detection’.

The method for silence detection is based on the idea that speech will yield a signal that is variable, while the absence of speech will result in a non-variable signal. First, cut-off parameters for a specific recording environment need to be determined. This was done for each session by calculating the absolute difference between signal maximum and signal minimum in small windows of 0.01 s. Windows with low signal variability will be associated with silence (small signal range). Looking at the distribution of all maximum to minimum distances in these windows in a histogram, one notices its positive skewness. This derives from the fact that windows containing silence will always yield the same (or highly similar) absolute maximum to minimum distances in their respective window and result in a high occurrence rate to the left of the histogram. Scott’s Rule was used to determine the adequate number of bins of the histogram to select a cut-off in terms of maximum to minimum variability. In a next step, this cut-off is applied to larger non-overlapping windows of 0.1 s. The selection of the right bin in the histogram was based on auditory probes. We found that this procedure was superior to any other available method that we tested on our dataset. The method is described in detail in the readme file and the manual [39]. The method has been used in preceding peer-reviewed publications [27, 28]. Based on the result of the procedure, start and stop times of the pauses relative to the start of the recording were extracted.

### 2.4. Classification of the speech pauses according to the speaker-switching patterns

Speaker diarisation is the process of determining who speaks when in recorded speech [40]. We used a supervised machine learning algorithm for this task. The following description of the procedure is slightly modified from study [27, p.162] in which the method was used on the current data set: A human scientific assistant extracted learning material, which was then used to train a machine learning algorithm to perform diarization of the complete material. If possible, the learning set was extracted for each dyad from two initial, two middle, and two final sessions. After this procedure, the learning set comprised a list of start and stop time stamps of samples of “patient speech” or “therapist speech” set with Audacity software [41]. We did not use transcripts. The learning set amounted to a minimum of 5-min of voice recordings per speaker. The features for machine learning were calculated in non-overlapping 0.2-s windows using a Matlab Audio Library [42]. This library computes 35 audio features for each window (e.g., mean fundamental frequency or Mel-frequency cepstral coefficients). The features and learning set were then used in a random forest classifier. This decision tree-based method learned to classify 0.2-s windows of patient or therapist speech based on the extracted features. The source code for this method can be retrieved from github [43]. Please consider the readme file for details. Furthermore, the procedure was described and validated on a speech corpus in a study by Fürer et al. [28] showing low error rates compared to unsupervised methods. The outcome of the procedure is the attribution of each detected speech utterance to either the psychotherapist or the patient in each dyad. In the current study, this information was used to classify each pause as belonging to one of four possible speaker switching patterns:

Patient speaks–Pause–Patient continues speaking (P_P); Patient speaks–Pause–Therapist speaks after the pause (P_T); Therapist speaks–Pause–Patient speaks (T_P); Therapist speaks–Pause–Therapist continues speaking (T_T).

For each of the patterns a pause variable was created, coding the proportion of the specific type of pauses.

### 2.5. Rupture coding

The “Rupture Resolution Rating System”, 3RS [7], was used to code ruptures and resolutions. The 3RS is an observer-based coding system to assess alliance rupture and resolution markers in psychotherapy. The 3RS differentiates between two types of ruptures: withdrawal and confrontation. It includes seven withdrawal markers, seven confrontation markers and ten resolution markers. A detailed definition of the rupture types and the markers can be found in the manual of the 3RS [7]. In addition, the 3RS provides a rupture significance rating using a five-point Likert scale ranging from *no significance* to *high significance*. It assesses the immediate impact that rupture markers inflict on the therapeutic alliance with respect to the impairment in collaboration regarding goals, tasks and the affective bond. The 3RS has demonstrated good interrater reliability (ICCs = .85 to .98) [44].

The rupture and resolution data for the current study is re-used from a study by Schenk et al. [22] who analysed 10 full psychotherapies. Additionally, the same team of trained observers rated the five first sessions of 13 additional patients using the same method. The rupture coding procedure has been described in the study by Schenk et al. [22]. Parts of the description below are re-used from this paper: The rupture and resolution detection and rating was done by two independent observers based on video recordings of the therapy sessions. The observers’ training involved reading the 3RS manual, training with a 3RS experienced research team from the Millennium Institute for Research in Depression and Personality (Santiago, Chile), and rating and discussing of exercise material. The observers were blind to the study hypotheses and patients’ diagnoses. The complete data collection is based on consensual coding according to a three-step qualitative procedure: i) Independent coding phase: each therapy session was rated independently by each observer; ii) Intersubjective consensus meeting: the two observers compared and re-evaluated their coding. If the observers did not achieve agreement, an observed event was marked for supervision; iii) Supervisor meeting: data collection was supervised by N. Schenk in monthly meetings in which unclear events were re-evaluated.

While the 3RS manual allows for episodes to belong to both rupture types, in the current study, the observers selected a dominant rupture type. Thus, rupture types were mutually exclusive in the current study. Additionally, all rupture markers were used. For withdrawal ruptures, the markers were: Denial, Minimal response, Abstract communication, Avoidant storytelling and/or shifting topic, Deferential and appeasing, Content/affect split, Self-criticism and/or hopelessness. For confrontation the markers were Complaints/concerns about the therapist, Patient rejects therapist intervention, Complaints/concerns about the activities of therapy, Complaints/concerns about the parameters of therapy, Complaints/concerns about progress in therapy, Patient defends self against therapist, Efforts to control/pressure therapist. The rupture markers are not mutually exclusive and as an episode can be coded as confrontation and withdrawal rupture at the same time, it is possible that the selected dominant type ended up with a marker belonging to the other type (e.g., a minimal response marker could appear in a confrontation withdrawal). In Schenk et al. [22], all markers were used. In the current study we only used the “minimal response” marker. Additionally, the rupture intensity was coded which is not relevant for the current study [7, 22]. While the 3RS mentions multiple techniques for delimiting markers (e.g., using a-priori fixed 5 minutes windows), for the current data, the observers marked the exact beginning and the end of rupture and resolution episodes and rated the observed rupture and resolution markers within these episodes.

### 2.6. Software and hardware

Data processing was done with R (v4.0.2) [45] for statistical computing. RStudio was used as the integrated development environment. We used the R packages lme4 (v1.1–28) [46], lmerTest (v3.1–3) [47] and sjPlot (v2.8.10) [48] for random effect models and their presentation; tidyverse (v1.3.1) [49] for data handling, ggplot (v3.3.5) [50] and RColorBrewer (v1.1–5) [51] for visualisations; data.table (v1.14.2) [52] for data writing and retrieving; and randomForest (v4.7–1) [53] and ROCR (v1.0–11) [54] for machine learning classification and performance evaluation. Calculations were performed at sciCORE (http://scicore.unibas.ch/) scientific computing centre at University of Basel.

### 2.7. Aggregation of pause and rupture data

Video time was divided into windows of 10 seconds length. For each window, the proportion of pauses compared to the total window length was calculated (sometimes, pauses could stretch over multiple windows which was then accounted for by splitting the pause episode proportional to their contribution to the different windows). Additionally, for each window, we determined whether it was part of a rupture episode or not. For each window marked as rupture, we also retained data specifying the rupture regarding the contained rupture markers ‘minimal response’ and the dominant type (withdrawal or confrontation).

### 2.8. Statistical analysis

Two linear mixed-effects models with random intercept by dyad were used to investigate differences in pauses according to rupture parameters. Percent of pauses in the 10 s windows was used as dependent variable in both models.

To test hypothesis H1 that ruptures are correlated with more speech pauses, Model A used ruptures vs. non-ruptures as predictor. Please see the formula of Model A in S1 File.

To test hypothesis H2 that withdrawal ruptures are characterised by more pauses than confrontation ruptures, Model B used rupture type as predictor. This model was exclusively based on rupture windows (excluding non-rupture windows). In order to additionally estimate the effect of the minimal response ruptures, presence of this marker was added as predictor as a fixed effect. P-values < 0.05 were considered statistically significant. As two mixed linear mixed-effects models were performed, we adjusted the p-values accordingly with the Bonferroni method. Please see the formula of Model B in S1 File.

**Fig 2:**
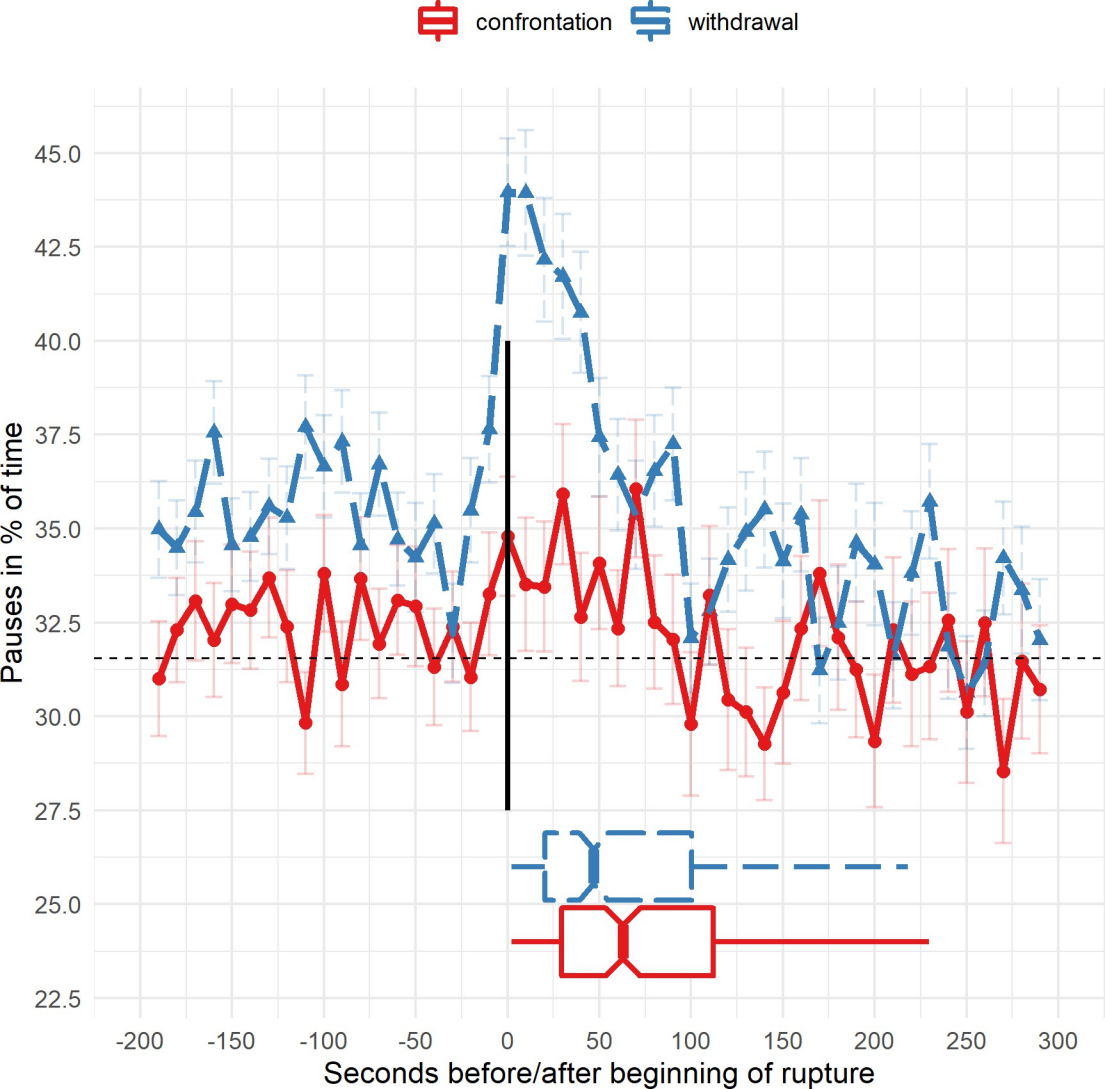
Pauses in the time course of ruptures–withdrawal vs. confrontation. Fig 2 shows the proportion of pauses during ruptures. To create this figure, the ruptures were overlapped at their starting point. The figure includes 20 windows (200 s) before and 30 windows (300 s) after the beginning of a rupture and the windows are overlapped relative to their distance from the starting point of the ruptures. The black vertical line at x = 0 indicates the starting point of the observed ruptures. Negative values on the x-axis represents the time before the ruptures. The boxplots at the bottom of the figure represent the length of the observed ruptures with the notch indicating the median end point of the ruptures to provide an impression of the rupture length in this figure. Each point (triangles for withdrawals, dots for confrontation) indicates the average of pauses in a 10 s window. Error bars indicate the standard error of mean for each window. The blue dotted line represents withdrawal ruptures and the red solid line confrontation ruptures. The black horizontal line shows average pauses across all windows.

For hypothesis H3 that minimal response ruptures can be predicted based on pauses and their speaker switching patterns, a random forests algorithm (RF) was used. RF is a machine learning classifier that uses decision trees [55, 56]. As an ensemble learner, the algorithm combines a certain amount of decision trees and uses them in a single prediction model [56]. RF is an algorithm known to easily obtain very good classification results while other approaches like deep learning might in some cases achieve better results when finetuned [57]. For a proof of principle study using RF seemed to be a sensible choice as it is known to perform well for many supervised classification problems while not requiring much finetuning. We used the guide by De Oliveira [58] for setting up the machine learning model. Faced with the problem of an imbalanced data set (only about 3% of the ten seconds windows contained minimal response marked ruptures), the RF was trained in rupture episodes only (discarding non-rupture windows). About 52% of the ruptures had a minimal response marker. We randomly selected 2/3 of the ruptures as a training set leaving 1/3 for validation. This selection was stratified according to presence of a minimal response marker and dyad and, additionally, weighted by the length of the ruptures in seconds. The predicted variable was minimal response (yes or no, in 10 s windows). The predicting features consisted of the z-transformed percentages (per dyad) of pauses according to the different speaker switching patterns (four variables: T_T, T_P, P_T, P_P) and additionally, those four variables lagged in both directions by 1–5 windows. For each window the model therefor ‘knew’ pause percentages 10, 20, 30, 40 and 50 seconds before and 10, 20, 30, 40, 50 seconds after the 10 s window in question. The approach was inspired by this blog [59]. These shifts were selected based on the information in Fig 2 which shows the timing of pauses related to ruptures. After training, we evaluated the obtained model on the validation set, and, additionally, on the validation set adding all the non-rupture windows (which were excluded during training). We used the out of the box settings provided by the ‘randomForest’ function in the randomForest R package (v4.7–1) [53]. We report the out-of-bag error as well as the importance measure for the features. Additionally, we calculated a receiver operator characteristic curve (ROC) i.e. plotting the true positive rate against the false positive rate [60, 61]. Finally, the classifier’s predictive accuracy was evaluated using the area under the curve (AUC), representing the probability that a random positive observation is ranked higher than a random negative observation [62]. Since the AUC considers the complete ROC curve with all possible classification thresholds, it is considered as a robust overall measure [63]. We omitted calculating cut-off scores as the analyses are meant as a proof of principle.

## 3. Results

In the 242 sessions stemming from the 22 psychotherapies, 221 confrontation ruptures and 295 withdrawal ruptures were identified by the observers. The average length of a confrontation rupture episode was 133.4 seconds (SD = 215.8). Withdrawal rupture episode had a length of 116.1 seconds (SD = 189.9). A total of 186 ruptures were marked with the ‘minimal response’ rupture marker of which 27 were found in confrontation ruptures and 159 in withdrawal ruptures.

The data was split into a total of 72’926 windows of 10 seconds. For each window, we know the percentage of pauses and whether it is a rupture or not. N = 6’870 of the windows were ruptures. Of those rupture windows, 3’183 were of the confrontation and 3’687 of the withdrawal dominant type and 3’564 included minimal response markers.

### 3.1. Pauses in ruptures (H1)

In non-ruptures, pauses made up on average 30.6% of the time (SD = 20%). In ruptures (confrontation and withdrawal taken together), pauses made up on average 41.5% of the time (SD = 24.8%). This difference (ruptures vs. non-ruptures) was statistically significant (p < 0.001) (Model A in the methods section). As the percent of pause variable presented with a slight positive skewness, we re-run the analysis with a square root transformed variable. The p-values remained highly significant.

### 3.2. Time course of pauses in relation to withdrawal vs. confrontation ruptures

Fig 2 shows the average time course of pauses in relation to the observer marked beginning of the 516 rupture episodes. The figure depicts a clear difference between the time course of pauses in withdrawal compared to confrontation ruptures. In confrontation ruptures, pauses appear to be only slightly increased compared to before the beginning of the ruptures. In withdrawal ruptures, a sharp increase of the proportion of pauses can be observed. Interestingly, the sharp increase begins 25 seconds before the observers marked the beginning of the ruptures. Even before this sharp increase, pauses are increased compared to the average (black broken horizontal line). After the ruptures ends, the proportion of pauses returns to the average (due to different lengths of the ruptures the timing of this normalisation is not exactly discernible in this summative figure).

A statistical test of the difference of withdrawal and confrontation ruptures is provided in the next section because it is a combined analysis of rupture type and the minimal response marker.

### 3.3. Speech pauses according to rupture type (H2) and presence of minimal response markers

Table 1 shows speech pause percentage according to the rupture type and minimal response marker.

Table 2 shows the results of the random effect model analysis (described as Model B in the methods section). Pauses in percent per 10 s window was predicted by the rupture type of the window and whether it contained a minimal response rupture marker.

**Table 2:** Percent of pauses predicted by rupture type and minimal response (Model B).

	**Percent of Pauses**						
*Predictors*	*Estimates*	*std*. *Error*	*std*. *Beta*	*std*. *std*. *Error*	*CI*	*std*. *CI*	*p*
(Intercept)	32.39	1.59	-0.34	0.06	29.27–35.52	-0.47–-0.21	**<0.001**
Rupture Type [withdrawal]	3.22	0.66	0.13	0.03	1.92–4.52	0.08–0.18	**<0.001**
Minimal response [yes]	11.67	0.77	0.47	0.03	10.17–13.18	0.41–0.53	**<0.001**
**Random Effects**							
σ^2^	512.38						
ICC	0.08						
N_Patient_	22						
Observations	6870						
Marginal R^2^ / Conditional R^2^	0.074 / 0.152						

Table 2 shows a random effect model with percent of pauses in 10 s windows as dependent variable. Rupture type and presence of minimal response markers in the corresponding window were use as predictors. Std. Error = Standard error of the estimate; std. Beta = standardised beta; std. std Beta = standardised standard error of the beta estimate; CI = confidence interval; std. CI = standardised confidence interval; p = p-value; ICC = intra class correlation; σ^2^ = random effect variance; Marginal R^2^ / Conditional R^2^ = coefficients of determination. The results differ from Table 1 because the model takes into account the contribution of the different therapeutic dyads.

According to the estimated effects of this model, windows belonging to confrontation ruptures without minimal responses have 32.39% of pauses. Compared to this value, withdrawal rupture windows present with 3.22% more pauses. Rupture windows with minimal response marker have 11.67% more pauses compared to confrontation ruptures without minimal response marker. The effects of rupture type are statistically significant (p < 0.001) with withdrawal rupture being correlated with a greater amount of pauses. This confirms hypothesis H2. Additionally, minimal response markers are statistically significantly associated with a greater amount of pauses (p < 0.001).

As the percent of pause variable presented with a slight positive skewness, we re-run the analysis with a square root transformed variable. The p-values remained highly significant.

### 3.4. Prediction of minimal response marked ruptures based on pauses (H3)

This section will use pauses in their respective speaker-switching patterns. S1 Table shows the percentages of pauses in these patterns in ruptures, non-ruptures, in withdrawal ruptures compared to confrontation ruptures and in minimal response marked ruptures compared to ruptures without this marker.

**Fig 3:**
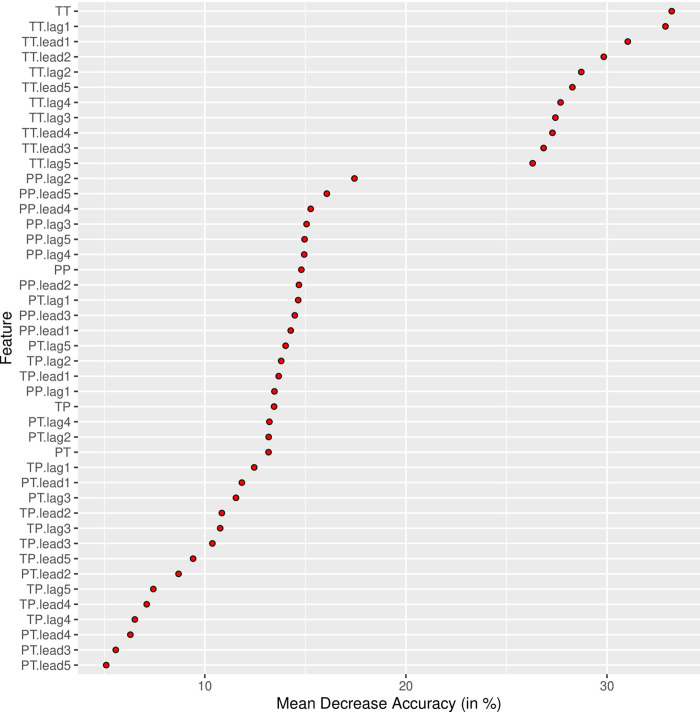
Pause-feature importance for the prediction of minimal response markers. Fig 3 shows the variable importance measure ‘Mean decrease accuracy’ for the predictive model. It indicates the loss of the model’s accuracy in percent if the variable in question is omitted from the training set. For an interpretation, please consider in which order the variables are ranked. The more important variables are listed at the top.

Training the random forest classifier to predict ruptures with minimal response markers with n = 500 trees and 6 variables tried at each split yielded an out of bag (OOB) estimate of error rate of 17.38%. Fig 3 presents the importance of the employed features (time lagged and lead pauses in different speaker-switching patterns). The trained model was published on Open Science Framework [64].

**Fig 4:**
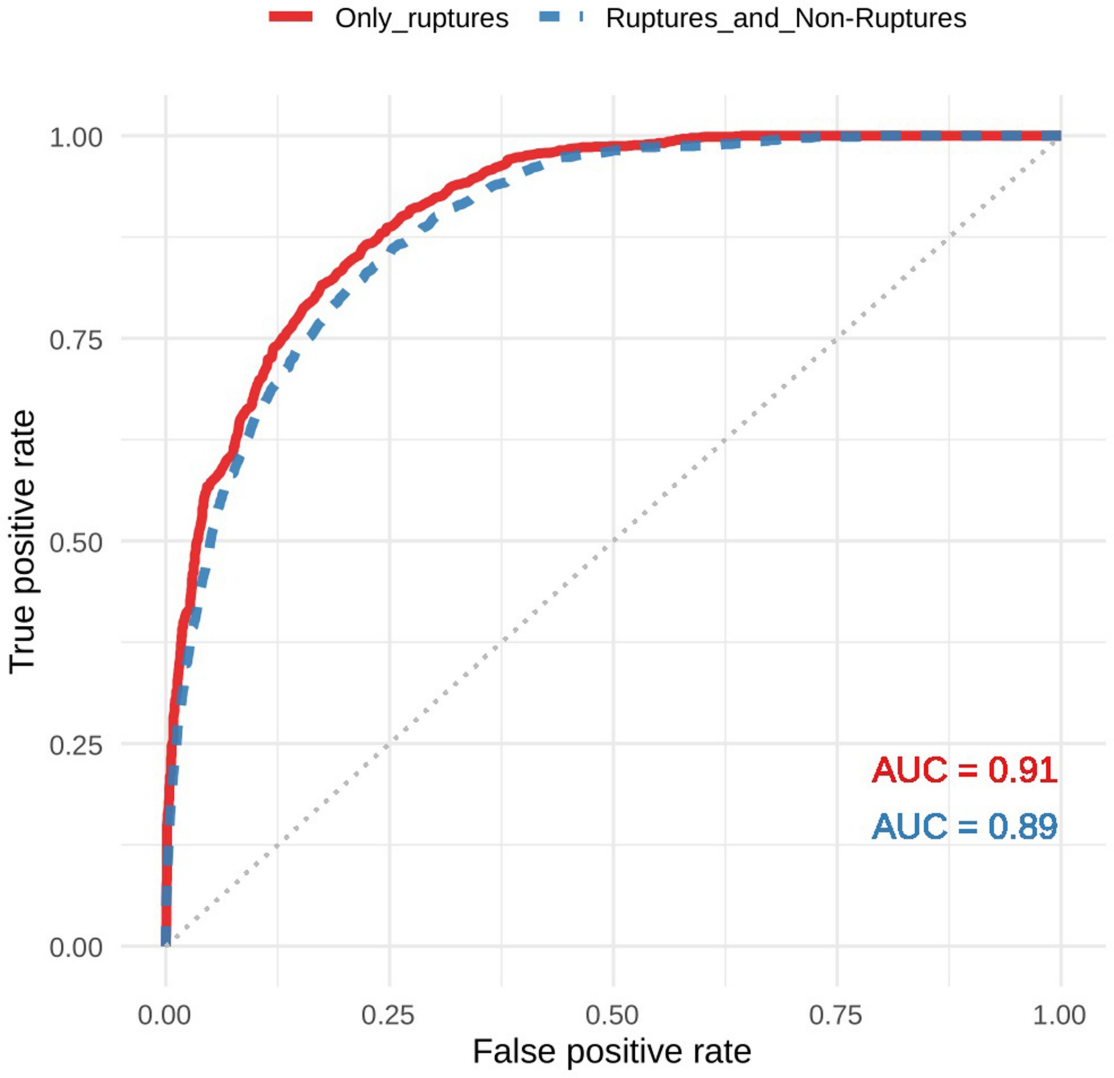
ROC for the prediction of minimal response markers in the validation data sets. Fig 4 shows the ROC for the minimal response marked rupture-prediction in the validation sets, constructed by plotting the true positivity rate against the false positive rate. The red line presents the performance in the validation set which only included ruptures. The dashed blue line presents performance in a validation set in which non-rupture data (by definition containing no minimal response markers) were added.

The feature importance estimation (Fig 3) showed that pauses belonging to the T_T speaker switching patterns and its lagged and lead versions show the highest importance for the RF model. Thus, pauses both lead and followed by therapist speech contain the most important information to identify minimal response marked ruptures. Using the trained RF model to predict ruptures with minimal response in the validation sets yielded 1) an AUC of 0.91 for the training set with only ruptures and 2) an AUC of 0.89 in the training set enriched with all 10-second windows that were not within a rupture. Fig 4 shows the ROC for both these predictions. These high AUC values confirm our hypothesis H3 which states that minimal response marked ruptures can be detected based on pauses.

### 3.5. Supplemental information regarding silence

Psychotherapy research has often used a cut-off of > = 3 seconds on pause data to address “silence” [23, 27]. The S2 and S3 Tables and S1–S3 Figs present the results as “silence” instead of “pauses”.

## 4. Discussion

As hypothesised (H1), ruptures (confrontation and withdrawal taken together) are correlated with more speech pauses. Further, we hypothesised that withdrawal ruptures would be characterised by more pauses than confrontation ruptures (H2). This hypothesis was clearly confirmed in adolescent patients with BPD by our results. This link between withdrawal ruptures and pauses exists independently from minimal-response markers. Minimal response markers (which are conceptually part of withdrawal ruptures) have an even stronger association with pauses, increasing the percentage of pauses by more than 10% (see Tables 1 and 2).

In an explorative analysis, the time course of pauses before, during and after ruptures was investigated (section 3.2, Fig 2). We found that pauses increase during withdrawal ruptures and this increase is observable even before the onset of the ruptures (see Fig 2). This is an interesting phenomenon with potential clinical applications (e.g., earlier use of repair strategies to prevent and train avoiding minimal response ruptures). At the end of the withdrawal ruptures, the proportion of pauses returns to an average level.

The hypothesis H3 (section 3.4) of the current paper stated that it would be possible to detect ruptures with the minimal response marker based on automatically extracted pause data and its location in the different speaker switching patterns (T_T, P_T, T_P, P_P, see section 2.4). We found that the prediction accuracy for minimal response marked ruptures performs at a level which allows to seriously consider this type of rupture localisation to facilitate psychotherapy research and enable psychotherapy quality management. The AUC of the ROC curve was 0.89. The variable importance of the machine learning model showed that pauses in the speaker switching pattern T_T (pause preceded and followed by therapist speech) was most predictive of minimal responses. This likely indicates that the psychotherapists are the ones carrying the dialogue during these episodes (the patient skips his/her speaking turns).

As discussed, methods for alliance rupture identification are currently mostly conducted manually and, thus, are time- and resource-consuming, and, they debatably lack reproducibility across laboratories [32, 65]. To overcome these limitations, the use of audio derived markers has been successfully attempted before [32], however, this attempt was limited to a case study. Here, we show that such detections are indeed feasible with a more general model across multiple dyads. However, it needs to be considered that the sample of patients in the current study was small (N = 22). A replication of the results in an independent sample is required and other disorders, age groups and psychotherapy approaches should be investigated to confirm the generalisability of our results. Additionally, we only predicted a specific rupture marker (minimal response). Aiming at minimal response marked ruptures made the target of the prediction more homogeneous. Another major difference to the study [32] was the usage of only one audio feature (pauses) as predictor. However, pauses have been exploited more deeply in the current study by using speaker switching patterns [43] and the time course of the feature (see Fig 2). In the previous study [32], withdrawal ruptures were, in addition to a higher pause proportion, associated with higher F0-span, shimmer, and lower articulation rate than neutral speech. Based on these results, it is possible that the performance of rupture detection can be improved and generalised to other rupture markers beyond what was shown in the current study. More emphasis on feature engineering will likely result in better performance regarding rupture localisation. The current study points towards the predictive importance of speaker switching patterns as well as the time dynamic of the features. Additionally, video-based features such as facial emotion recognition [29] or motion energy analysis [30] have the potential to improve the predictive performance beyond what can be achieved with audio features alone. Finally, predictive features can be engineered to represent the interaction of the patient and the therapist (e.g., synchrony measures) [66–68] which might potentially enable the detection of confrontation and withdrawal ruptures or other significant episodes e.g., moments of psychotherapeutic change [69].

## Supporting information

S1 File Formulas of the random mixed effect models (Model A and Model B). (PDF)

S1 Table Description of pause percentage in speaker-switching patterns. This table describes the percentage of pauses in the different speaker switching patterns for rupture and non-rupture events, withdrawal and confrontation ruptures, as well as for minimal response marked ruptures and ruptures without this marker. *Mdn* = Median; *Q1* = 1st quartile; *Q3* = 3rd quartile; *M* = arithmetic mean; *SD* = standard deviation.
(DOCX)

S2 Table 3 Seconds filter–percent of silence in rupture and non-rupture events. S3 shows the percent of silence in rupture and non-rupture events, confrontation and withdrawal ruptures, as well as minimal response marked ruptures and ruptures without minimal response marker, when the 3 s filter for silence is applied. *Mdn* = Median; *Q1* = 1st quartile; *Q3* = 3rd quartile; *M* = arithmetic mean; *SD* = standard deviation.
(DOCX)

S3 Table 3 Seconds filter—description of silence percentage in speaker-switching patterns. This table describes the percentage of silence in the different speaker switching patterns for rupture and non-rupture events, withdrawal and confrontation ruptures, as well as for minimal response marked ruptures and ruptures without this marker, when the 3 s filter for silence episodes is applied. *Mdn* = Median; *Q1* = 1st quartile; *Q3* = 3rd quartile; *M* = arithmetic mean; *SD* = standard deviation.
(DOCX)

S1 Fig 3 Seconds filter–silence in the time course of ruptures. S5 shows the proportion of pauses during ruptures, when the 3 s filter for silence is applied. Please consider the legend in Fig 2 for further information on the figures’ creation.
(PDF)

S2 Fig 3 Seconds filter–silence-feature importance for the prediction of minimal response markers. S6 shows the variable importance measure ‘Mean decrease accuracy’ for the predictive model when the 3 s silence filter is applied. It indicates the loss of the model’s accuracy in percent if the variable in question is omitted from the training set. For an interpretation, please consider in which order the variables are ranked. The more important variables are listed at the top.
(PDF)

S3 Fig 3 Seconds filter–roc for the prediction of minimal response markers in the validation data sets. This figure shows the ROC for the minimal response marked rupture-prediction in the validation sets, constructed by plotting the true positivity rate against the false positive rate, when the 3 s silence filter is applied. The red line presents the performance in the validation set which only included ruptures. The dashed blue line presents performance in a validation set in which non-rupture data (by definition containing no minimal response markers) were added.
(PDF)
