# Supplementary material for: Withdrawal ruptures in adolescents with borderline personality disorder psychotherapy are marked by increased speech pauses–can minimal responses be automatically detected?
Source: PLoS One. 2023 Jan 17;18(1):e0280329. doi: 10.1371/journal.pone.0280329 (PMC9844899; doi:10.1371/journal.pone.0280329)
Supplement: S1 File — (PDF) [file pone.0280329.s001.pdf]

Formulas for ModelA and ModelB

2022-09-10

Formula for Model A, testing hypothesis H1

$$\begin{aligned} \text{Silence\_Length\_Percent}_i &\sim N\left(\alpha_{j[i]} + \beta_1(\text{Rupture}_{no}), \sigma^2\right) \\ \alpha_j &\sim N\left(\mu_{\alpha_j}, \sigma^2_{\alpha_j}\right), \text{ for Patient j = 1, ... ,J} \end{aligned}$$

Formula for Model B, testing hypothesis H2

$$\begin{aligned} \text{Silence\_Length\_Percent}_i &\sim N\left(\alpha_{j[i]} + \beta_1(\text{Rupture. Type}_{\text{withdrawal}}) + \beta_2(\text{MinimalResponse}_{\text{No}}), \sigma^2\right) \\ \alpha_j &\sim N\left(\mu_{\alpha_j}, \sigma^2_{\alpha_j}\right), \text{ for Patient j = 1, ... ,J} \end{aligned}$$
