# Supplementary material for: Withdrawal ruptures in adolescents with borderline personality disorder psychotherapy are marked by increased speech pauses–can minimal responses be automatically detected?
Source: PLoS One. 2023 Jan 17;18(1):e0280329. doi: 10.1371/journal.pone.0280329 (PMC9844899; doi:10.1371/journal.pone.0280329)
Supplement: S1 Table — This table describes the percentage of pauses in the different speaker switching patterns for rupture and non-rupture events, withdrawal and confrontation ruptures, as well as for minimal response marked ruptures and ruptures without this marker. Mdn = Median; Q1 = 1st quartile; Q3 = 3rd quartile; M = arithmetic mean; SD = standard deviation. (DOCX) [file pone.0280329.s002.docx]

|  | **Percent of Pauses (P_P)** | | | | | |
| --- | --- | --- | --- | --- | --- | --- |
| *Rupture* | *Mdn* | *Q1* | *Q3* | *M* | *SD* |  |
| No Rupture | 16.0 | 8.0 | 26.0 | 19.3 | 15.2 |  |
| Rupture | 14.0 | 8.0 | 26.0 | 19.7 | 17.2 |  |
| Confrontation Rupture | 14.0 | 8.0 | 26.0 | 18.6 | 15.8 |  |
| Withdrawal Rupture | 16.0 | 8.0 | 28.0 | 21.1 | 18.7 |  |
| No Minimal Response | 14.0 | 8.0 | 26.0 | 19.4 | 15.5 |  |
| Minimal Response | 16.0 | 6.0 | 26.0 | 20.5 | 20.5 |  |
|  |  | | | | | |

|  | **Percent of Pauses (P_T)** | | | | | |
| --- | --- | --- | --- | --- | --- | --- |
| *Rupture* | *Mdn* | *Q1* | *Q3* | *M* | *SD* |  |
| No Rupture | 10.0 | 6.0 | 20.0 | 16.3 | 16.2 |  |
| Rupture | 12.0 | 6.0 | 26.0 | 19.6 | 19.9 |  |
| Confrontation Rupture | 10.0 | 6.0 | 22.0 | 17.1 | 17.2 |  |
| Withdrawal Rupture | 14.0 | 6.0 | 30.0 | 21.7 | 21.7 |  |
| No Minimal Response | 12.0 | 6.0 | 24.0 | 17.6 | 17.3 |  |
| Minimal Response | 14.0 | 6.0 | 30.0 | 21.8 | 22.3 |  |
|  |  | | | | | |

(S1 **Continuation)**

|  | **Percent of Pauses (T_P)** | | | | | |
| --- | --- | --- | --- | --- | --- | --- |
| *Rupture* | *Mdn* | *Q1* | *Q3* | *M* | *SD* |  |
| No Rupture | 10.0 | 6.0 | 18.0 | 14.6 | 14.4 |  |
| Rupture | 12.0 | 6.0 | 22.0 | 17.9 | 18.4 |  |
| Confrontation Rupture | 10.0 | 6.0 | 18.0 | 15.6 | 16.9 |  |
| Withdrawal Rupture | 14.0 | 6.0 | 26.0 | 19.8 | 19.4 |  |
| No Minimal Response | 10.0 | 6.0 | 20.0 | 15.4 | 15.4 |  |
| Minimal Response | 12.0 | 6.0 | 26.0 | 20.2 | 20.5 |  |
|  |  | | | | | |

|  | **Percent of Pauses (T_T)** | | | | | |
| --- | --- | --- | --- | --- | --- | --- |
| *Rupture* | *Mdn* | *Q1* | *Q3* | *M* | *SD* |  |
| No Rupture | 18.0 | 10.0 | 34.0 | 23.9 | 19.7 |  |
| Rupture | 24.0 | 12.0 | 44.0 | 31.6 | 25.1 |  |
| Confrontation Rupture | 20.0 | 10.0 | 38.0 | 27.2 | 26.3 |  |
| Withdrawal Rupture | 28.0 | 14.0 | 50.0 | 34.8 | 26.3 |  |
| No Minimal Response | 18.0 | 8.0 | 32.0 | 23.2 | 19.0 |  |
| Minimal Response | 30.0 | 14.0 | 54.0 | 36.7 | 26.9 |  |
|  |  | | | | | |
