# Supplementary material for: Withdrawal ruptures in adolescents with borderline personality disorder psychotherapy are marked by increased speech pauses–can minimal responses be automatically detected?
Source: PLoS One. 2023 Jan 17;18(1):e0280329. doi: 10.1371/journal.pone.0280329 (PMC9844899; doi:10.1371/journal.pone.0280329)
Supplement: S2 Table — S3 shows the percent of silence in rupture and non-rupture events, confrontation and withdrawal ruptures, as well as minimal response marked ruptures and ruptures without minimal response marker, when the 3 s filter for silence is applied. Mdn = Median; Q1 = 1st quartile; Q3 = 3rd quartile; M = arithmetic mean; SD = standard deviation. (DOCX) [file pone.0280329.s003.docx]

|  | **Percent of Silence** | | | | | |
| --- | --- | --- | --- | --- | --- | --- |
| *Rupture* | *Mdn* | *Q1* | *Q3* | *M* | *SD* |  |
| No Rupture | 0 | 0 | 10.0 | 11.0 | 22.0 |  |
| Rupture | 0 | 0 | 38.0 | 21.1 | 29.4 |  |
| Confrontation Rupture | 0 | 0 | 28.0 | 15.4 | 25.6 |  |
| Withdrawal Rupture | 8.00 | 0 | 48.0 | 26.0 | 31.6 |  |
| No Minimal Response | 0 | 0 | 20.0 | 12.7 | 23.1 |  |
| Minimal Response | 18.0 | 0 | 52.0 | 28.9 | 32.3 |  |
|  |  | | | | | |
