# Supplementary material for: Withdrawal ruptures in adolescents with borderline personality disorder psychotherapy are marked by increased speech pauses–can minimal responses be automatically detected?
Source: PLoS One. 2023 Jan 17;18(1):e0280329. doi: 10.1371/journal.pone.0280329 (PMC9844899; doi:10.1371/journal.pone.0280329)
Supplement: S3 Table — This table describes the percentage of silence in the different speaker switching patterns for rupture and non-rupture events, withdrawal and confrontation ruptures, as well as for minimal response marked ruptures and ruptures without this marker, when the 3 s filter for silence episodes is applied. Mdn = Median; Q1 = 1st quartile; Q3 = 3rd quartile; M = arithmetic mean; SD = standard deviation. (DOCX) [file pone.0280329.s004.docx]

|  | **Percent of Silence (P_P)** | | | | | |
| --- | --- | --- | --- | --- | --- | --- |
| *Rupture* | *Mdn* | *Q1* | *Q3* | *M* | *SD* |  |
| No Rupture | 32.0 | 20.0 | 42.0 | 33.8 | 21.1 |  |
| Rupture | 34.0 | 20.0 | 48.0 | 36.4 | 22.1 |  |
| Confrontation Rupture | 34.0 | 20.0 | 46.0 | 35.1 | 21.0 |  |
| Withdrawal Rupture | 34.0 | 21.0 | 50.0 | 37.5 | 23.0 |  |
| No Minimal Response | 32.0 | 18.0 | 44.0 | 33.2 | 19.8 |  |
| Minimal Response | 36.0 | 22.5 | 56.0 | 41.2 | 24.5 |  |
|  |  | | | | | |

|  | **Percent of Silence (P_T)** | | | | | |
| --- | --- | --- | --- | --- | --- | --- |
| *Rupture* | *Mdn* | *Q1* | *Q3* | *M* | *SD* |  |
| No Rupture | 34.0 | 20.0 | 46.0 | 35.9 | 22.6 |  |
| Rupture | 34.0 | 20.0 | 52.0 | 38.6 | 24.6 |  |
| Confrontation Rupture | 34.0 | 19.5 | 50 | 36.3 | 23.0 |  |
| Withdrawal Rupture | 34.0 | 22.0 | 52.0 | 40.0 | 25.5 |  |
| No Minimal Response | 34.0 | 20.0 | 50.0 | 36.2 | 22.0 |  |
| Minimal Response | 36.0 | 20.0 | 54.0 | 40.6 | 26.2 |  |
|  |  | | | | | |

(S3 **Continuation)**

|  | **Percent of Silence (T_P)** | | | | | |
| --- | --- | --- | --- | --- | --- | --- |
| *Rupture* | *Mdn* | *Q1* | *Q3* | *M* | *SD* |  |
| No Rupture | 32.0 | 20.0 | 46.0 | 35.3 | 22.1 |  |
| Rupture | 34.0 | 18.0 | 50.0 | 37.1 | 24.8 |  |
| Confrontation Rupture | 32.0 | 16.0 | 44.5 | 34.7 | 24.8 |  |
| Withdrawal Rupture | 34.0 | 22.0 | 52.0 | 38.7 | 24.7 |  |
| No Minimal Response | 32.0 | 18.0 | 44.0 | 34.0 | 22.6 |  |
| Minimal Response | 34.0 | 20.0 | 54.0 | 39.0 | 25.8 |  |
|  |  | | | | | |

|  | **Percent of Silence (T_T)** | | | | | |
| --- | --- | --- | --- | --- | --- | --- |
| *Rupture* | *Mdn* | *Q1* | *Q3* | *M* | *SD* |  |
| No Rupture | 34.0 | 22.0 | 52.0 | 38.6 | 24.3 |  |
| Rupture | 40.0 | 24.0 | 62.0 | 44.3 | 27.8 |  |
| Confrontation Rupture | 36.0 | 20.0 | 54.0 | 39.9 | 26.4 |  |
| Withdrawal Rupture | 42.0 | 26.0 | 66.0 | 46.6 | 28.3 |  |
| No Minimal Response | 32.0 | 18.0 | 48.0 | 35.6 | 23.7 |  |
| Minimal Response | 42.0 | 26.0 | 66.0 | 46.8 | 28.4 |  |
|  |  | | | | | |
