# Supplementary figures and images for: Withdrawal ruptures in adolescents with borderline personality disorder psychotherapy are marked by increased speech pauses–can minimal responses be automatically detected?
Source: PLoS One. 2023 Jan 17;18(1):e0280329. doi: 10.1371/journal.pone.0280329 (PMC9844899; doi:10.1371/journal.pone.0280329)

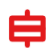 confrontation 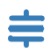 withdrawal

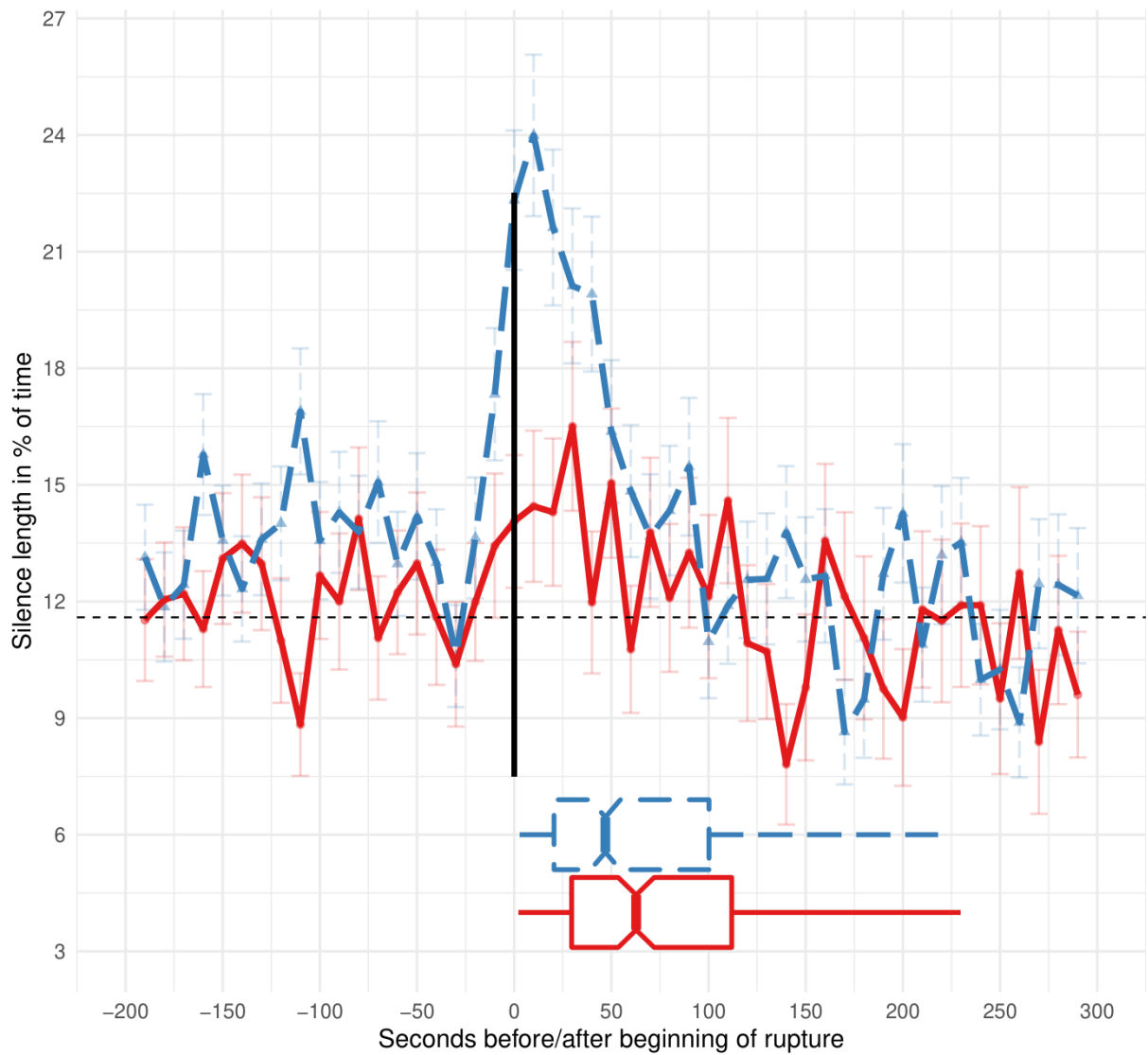

Supplement: S1 Fig — S5 shows the proportion of pauses during ruptures, when the 3 s filter for silence is applied. Please consider the legend in Fig 2 for further information on the figures’ creation. (PDF) [file pone.0280329.s005.pdf]

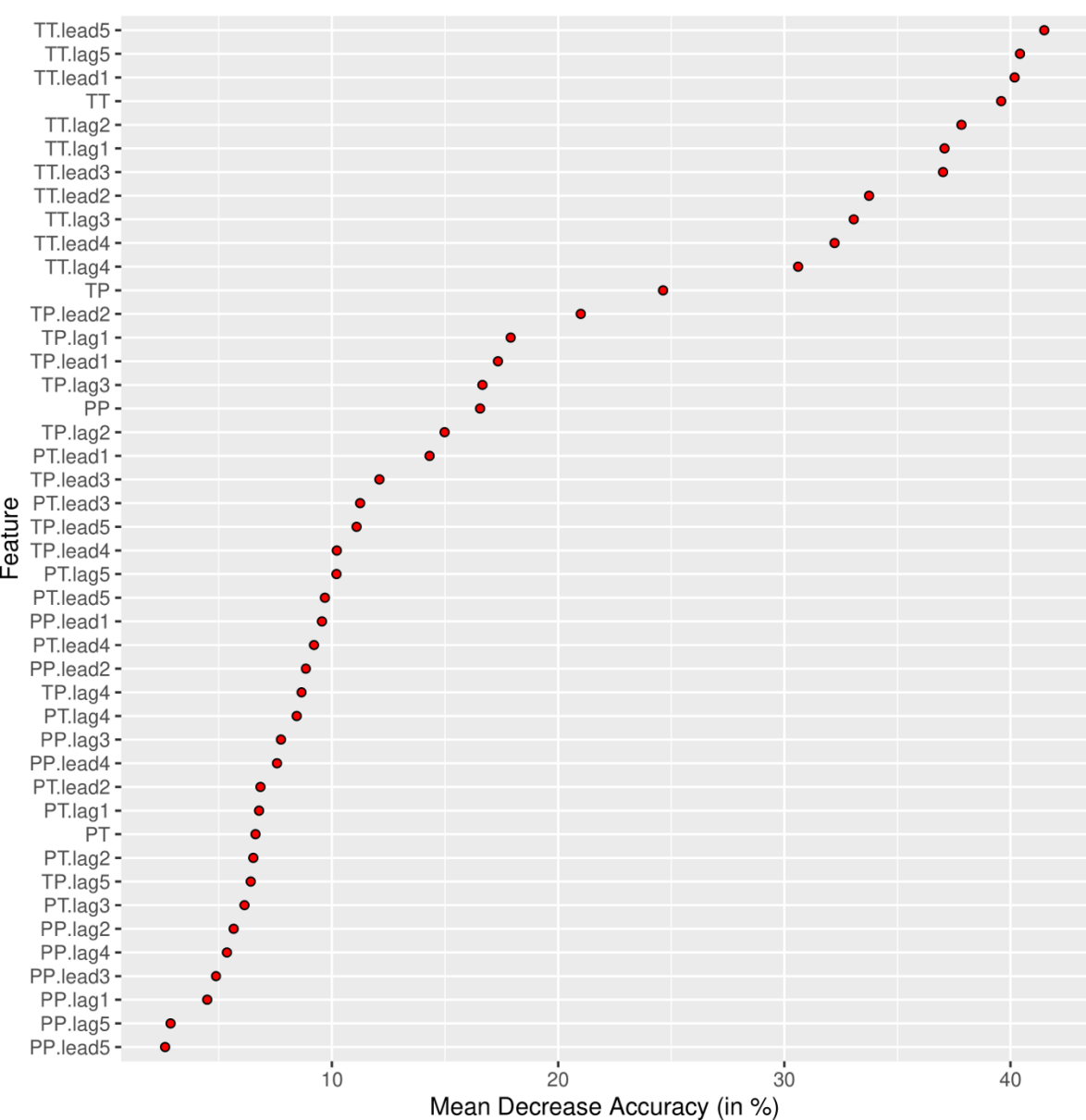

Supplement: S2 Fig — S6 shows the variable importance measure ‘Mean decrease accuracy’ for the predictive model when the 3 s silence filter is applied. It indicates the loss of the model’s accuracy in percent if the variable in question is omitted from the training set. For an interpretation, please consider in which order the variables are ranked. The more important variables are listed at the top. (PDF) [file pone.0280329.s006.pdf]

True positive rate

Only\_ruptures Ruptures\_and\_Non-Ruptures

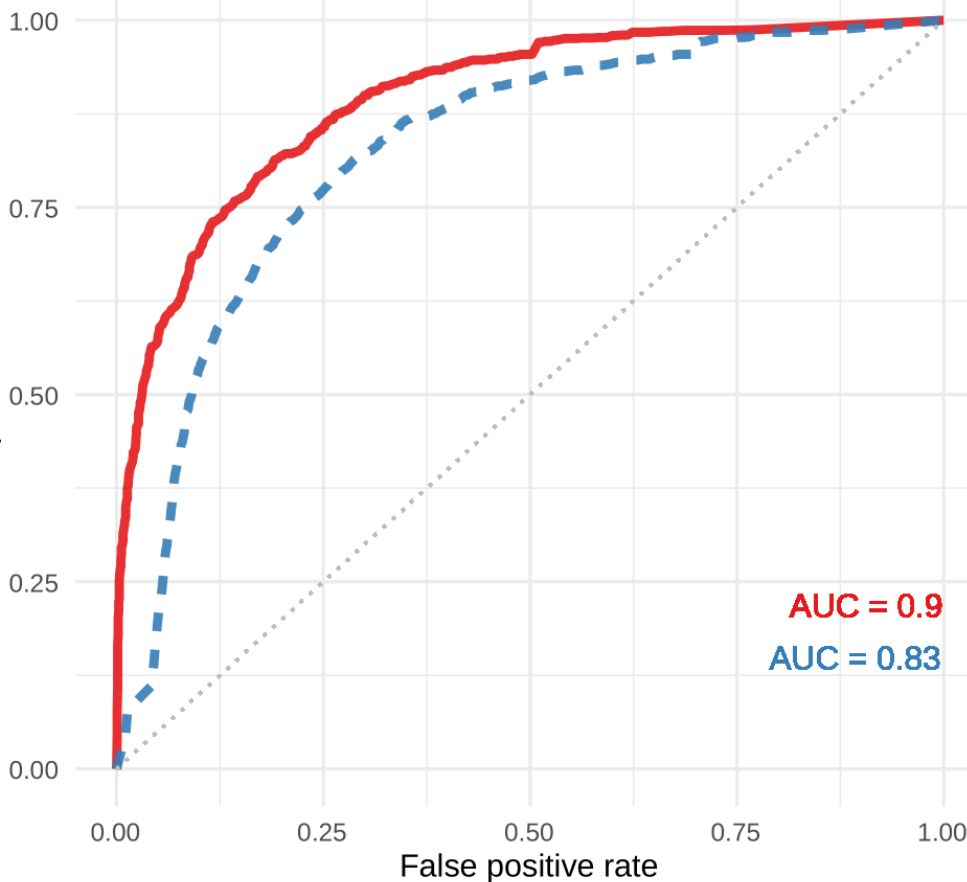

Supplement: S3 Fig — This figure shows the ROC for the minimal response marked rupture-prediction in the validation sets, constructed by plotting the true positivity rate against the false positive rate, when the 3 s silence filter is applied. The red line presents the performance in the validation set which only included ruptures. The dashed blue line presents performance in a validation set in which non-rupture data (by definition containing no minimal response markers) were added. (PDF) [file pone.0280329.s007.pdf]
